# Mental health challenges during COVID-19: perspectives from parents with children with neurodevelopmental disabilities

**DOI:** 10.1080/17482631.2022.2136090

**Published:** 2022-10-30

**Authors:** Genevieve Currie, Brittany Finlay, Ashish Seth, Christiane Roth, Myada Elsabbagh, Anne Hudon, Matthew Hunt, Sebastien Jodoin, Lucyna Lach, Raphael Lencucha, David B. Nicholas, Keiko Shakako, Jennifer Zwicker

**Affiliations:** a School of Public Policy, University of Calgary, Calgary, AB, Canada; b School of Nursing and Midwifery, Mount Royal University, Calgary, AB, Canada; c Faculty of Medicine, McGill University, Montreal, Que., Canada; d Faculty of Medicine, Universite de Montreal, Montreal, Que., Canada; e Faculty of Medicine and Health Sciences School of Physical and Occupational Therapy, McGill University, Montreal, Que., Canada; f Faculty of Law, McGill University, Montreal, Que., Canada; g School of Social Work, McGill University, Montreal, Que., Canada; h Faculty of Social Work, University of Calgary, Calgary, AB, Canada; i School of Public Policy; Faculty of Kinesiology, University of Calgary, Calgary, AB, Canada

**Keywords:** Parents, children, youth, mental health, COVID-19, disabilities

## Abstract

**Background:**

The global pandemic and subsequent denials, delays, and disruptions in essential daily activities created significant challenges for children with neurodevelopmental disorders (NDDs) and their parents. Public health restrictions during the COVID-19 pandemic limited access to supports and services required by children with NDDs to maintain their health and well-being.

**Objective:**

This study sought to understand the impacts of these public health measures and restrictions on mental health from the perspective of parents with children with NDDs to inform pathways for public health policies responsive to the needs of this population.

**Method:**

Interpretive descriptive design was used to guide data collection and data analysis. Forty caregivers were interviewed about their experience with pandemic restrictions.

**Findings:**

Generic policy measures contributed to many gaps in families’ social support systems and contributed to mental health challenges for children and their parents. Four themes emerged: 1) lack of social networks and activities, 2) lack of access to health and social supports, 3) tension in the family unit, and 4) impact on mental health for children and their parents.

**Recommendations:**

Emergency preparedness planning requires a disability inclusive approach allocating resources for family supports in the home and community. Families identified supports to minimize further pandemic disruptions and enhance recovery.

## Introduction

The global pandemic and subsequent denials, delays, and disruptions in essential daily activities such as school, work, and community living created significant challenges for children and youth with neurodevelopmental disabilities (NDDs) and their parents. Children and youth with NDDs require particular consideration to ensure their continued participation in everyday life (Rosenbaum & Novak-Pavlic, 2021). This population requires targeted supports during a public health emergency. It was particularly challenging when public health restrictions created during the COVID-19 pandemic limited access to basic structures, services, and supports required by families and youth with NDDs to maintain their health and well-being. This study sought to understand the impacts of these public health measures and restrictions on mental health from the perspective of parents with children and youth. These insights will be used to inform pathways for public health policies that are responsive to the needs of this population.

### Children with NDDs and support systems

Globally, there were 52.8 million children with a NDD in 2016 (Olusanya et al., 2018). In Canada, 10 to 17% of children have a NDD and 90% of these children require supports to participate in daily activities of living (”Government of Canada,” 2020). Children with NDDs often have chronic comorbidities affecting the brain and neurological system from genetic and environmental factors. These comorbidities could lead to cognitive and behavioural challenges, and limited capacity in social skills, communication, and self-care (Einfeld et al., 2011; Tassé et al., 2012). Children with NDDs often require integration of social support from systems, social structures, and policies that facilitate their participation and citizenship in school, play, and daily life activities (Bach, 2017). Children may also access multiple community-based health care, educational, and social services (Cohen et al., 2011). These supports include nursing services, developmental, educational, and behavioural aides, therapies, and respite care. Parents often serve as the primary caregivers for their children, providing and coordinating the services and social supports to their children at home and in the community (Gardiner et al., 2018; Grumi et al., 2021).

### Mental health implications of COVID measures

During the pandemic many services and activities were denied or disrupted, including many school-based supports. Social policies to limit the spread of disease were enforced at different phases of the pandemic, including physical distancing measures in public spaces, restrictions on group gatherings, and limited or denied access in supports in the home from extended family members or services providers (Canadian Public Health Association, 2021). Health and rehabilitation services that were not considered essential were also initially cancelled and eventually adapted to virtual appointments with many medical specialists and counsellors and therapists (Vogel, 2020). Services such as physical or occupational/speech therapies were often postponed or cancelled for many months and were not offered with remote platforms as many required in-person attention from the therapist and could not be safely executed by non-trained individuals. Together these delays and disruptions may have had wide ranging implications for the participation of children in activities of daily life, contributing to mental health challenges faced by this group.

Before the pandemic, children with NDDs had a disproportionately higher incidence of mental health challenges than the general population (Solmi et al., 2021). For example, studies demonstrated that autistic children had a twofold higher rate of anxiety than their neurotypical peers (Van Steensel et al., 2011) and a four times higher incidence of depression (Hudson et al., 2019). Children with neurodiversity were also more susceptible to mental health effects with the pandemic according to Samji et al. (2021) in a systematic review of the literature. Increases in mental health conditions during the pandemic such as anxiety, distress, depression, and worsening of symptoms for pre-existing conditions have been noted for children with NDD in research studies across the world (Breaux et al., 2021; Gadermann et al., 2021; Guller et al., 2021; Shorey et al., 2021; Zhang et al., 2020). Also noted in the literature was a worsening of symptoms if mental health supports were restricted or denied (Moreno et al., 2020).

Parents of children with NDDs also experienced higher rates of depression and anxiety before the pandemic (Bayer et al., 2021; McCann et al., 2016; Scherer et al., 2019). During the pandemic, mental health implications for families caring for children with NDDs were also documented (Bentenuto et al., 2021; Cluver et al., 2020; Gadermann et al., 2021; Guller et al., 2021; Lee et al., 2021; Patrick et al., 2020; Shorey et al., 2021; Wang et al., 2021). The literature demonstrated that children’s mental health was impacted, though studies also showed an increased in resilience and behaviour for some children, related to reduced outings, simpler routines, and less transitions during lockdown and confinement (Yusuf et al., 2022). Despite these studies, there is still a limited understanding of how specific policies and pandemic restrictions impacted the mental health and overall functioning of children with NDDs and their parents in different regions and jurisdictions. The pandemic response and restrictions were different in each country. This study highlighted restrictions, policies, and timelines that were relevant for jurisdictions in Canada.

The social determinants of health and right based frameworks, support an understanding of contextual factors such as policies, socio-cultural environment, and systems of care which influence mental health and distress (Alegría et al., 2018; Bach, 2017; Filipe et al., 2021) Obtaining a better understanding of perspectives from caregivers of children and youth with NDD on how Canadian government policies and restrictions from the COVID-19 pandemic affected their day-to-day participation in society can help create policies and programmes that are more responsive to the needs of this population in the upcoming recovery process and future public health emergencies. Better understanding of families’ experiences could also strengthen mental health supports for children with NDDs and their caregivers. The objective of this study was to examine the impact of COVID-19 policy measures in Canada on the mental health of parents/caregivers (hereafter referred to as parents) of children and youth (hereafter referred to as children) with NDDs. This knowledge will be used to inform disability inclusive policy approaches for children with NDDs and their parents.

## Methodology

### Design

We adopted a qualitative design using Interpretive Description (ID; Thorne, 2008). ID is a qualitative research approach used to explore critical problems within an applied area to inductively describe and to offer interpretive explanations of a phenomena that has applied implications (Thorne et al., 1997, 2004). This qualitative description approach was used to capture and describe mental health impacts and experiences during the COVID-19 pandemic (Bradshaw et al., 2017) and to obtain rich data to inform disability inclusive policy approaches (Sullivan-Bolyai et al., 2005). ID was also used to guide synthesis of participants’ perspectives and experiences in the development of synopses after each interview (Thompson Burdine et al., 2021). The study was approved by the xx Conjoint Faculties Research Ethics Board (REB17-1585).

### Recruitment and sample

Parents of children with NDDs aged 0 to 30 years of age were approached by our research team. A sample of 81 parents had been recruited for a previous study investigating provincial and federal disability programmes. These parents consented to be contacted for future studies for rapid recruiting and advancing analysis and impact (See xx for further information about the original population sample). A purposive maximum variation sampling strategy (Sullivan-Bolyai et al., 2005) was used to recruit participants across age, gender, and severity/type of NDDs, family income, province/territory of residence, rural/urban home location, and Indigenous status to ensure diverse perspectives. Forty parents agreed to be interviewed in this study. Variability across provinces and territories was sought so parents were separated into their provinces/territories of residence. For provinces that had larger representation, variability was sought with other demographics. Interviews continued until all participants from the larger pool of 81 parents were contacted. For underrepresented provinces in Atlantic Canada and the Yukon, recruitment support was sought from our transdisciplinary research team and advisory council to increase recruitment in underrepresented regions. Data richness was obtained from the diversity of perspectives gained from maximum variation sampling. The majority of participants who consented to participate in the study were female as they identified themselves as the primary caregivers. This is consistent with the literature that women are most often the primary caregivers of children with disabilities (Douglas et al., 2021 Knudson‐Martin & Silverstein, 2009; Runswick-Cole & Ryan, 2019) including children with NDDs (Kayadjanian et al., 2018; Lanfranchi & Vianello, 2012).

### Data collection

Prior to commencing semi-structured interviews, verbal or written informed consent was obtained. The study involved interviews with parents/caregivers of children with NDDs in both English and French. Participation was confidential and data collection procedures insured anonymity. Participants could withdraw from the study at any time. Interviews were conducted between February 2021 and August 2021 during the height of the third wave of the COVID-19 pandemic in Canada. Interviews were audio recorded and took place over a secure internet platform or by telephone. Participants were offered a $25.00 gift card after participation in the study.

The interview guide was pilot tested by a family research partner and modifications were made. The guide was then reviewed by a transdisciplinary research team and advisory council. The research team and advisory council consisted of persons or family members living with NDDs, knowledge users, community partners, policy makers, and disability rights experts. Interview questions centred on access to services, employment, education, precautionary health measures, and mental health (See Appendix 1 for the interview guide). Participants responded to open-ended questions describing their mental health challenges, needs, and recent experiences of caregiving for children with disabilities across provinces/territories during the COVID-19 pandemic Prompts included requests to describe experiences of their immediate family members and children with NDD during the COVID-19 pandemic and impacts if any on their own mental health and that of their children. Other impacts on parents with children with NDDs regarding income, employment, educational supports, precautionary measures, and access to services during the pandemic are published elsewhere (Seth et al., 2022).

### Data analysis

Data collection and analysis occurred sequentially. English interviews were transcribed verbatim using Rev transcription software. Transcripts were reviewed for accuracy by the research team. French interviews were transcribed verbatim by a professional translation company, Intersigne and then translated into English by a bilingual team member for analysis. Thematic analysis (Braun & Clarke, 2006) was used to analyse the interview data into categories and codes. The research investigators (JZ, DN, LL, KT, MH, AP, RL, ME, RL, SJ) supported the coders (GC, AS, BF) throughout the data analysis process. After analysing three interview transcripts separately and then together, an initial codebook was created by the research team (BF, GC, AS) for each category and code. The codebook contained labelled codes and definitions for each code. Three more interviews were subsequently coded to assess if the codebook required any additional or revised categories. Through weekly meetings, the team updated the codebook by adding and refining codes. The codes were clustered and themes were developed inductively by the team (BF, GC, AS, CR) based on the research question. Descriptive analysis was used in interactive discussion with the entire research team, to expand the understanding of the phenomena and to identify practical implications of the analysis, which were incorporated into the themes and subsequent analysis steps. To achieve consistency with coding, the research team and coders met regularly and discussed the analysis. Key messages were developed from the themes and sub themes and included variations and similarities in parents’ perspectives. Each interview was synthesized into a synopsis providing an overview of the participants’ backgrounds, topics discussed, and important experiences. Team members (MH, RL, AH, LL, DN, JZ) and advisory council members reviewed and provided feedback on the initial synopses. The synopses provided a summary of the interview including key topics, issues, or experiences for feedback and reflections during the analysis process from the research team and advisory council members. The synopses summarized data related to the research objectives and discerned similarities and distinctions between participant experiences (Thompson Burdine et al., 2021). NVivo12 software was used to store and organize the data. Rigour was supported by maintaining an audit trail of analysis decisions, communicating analysis to the advisory council, and broader research team and research participants by sharing the initial findings in a summary document for feedback.

## Results

A total of forty (40) parents from across Canada, representing all provinces and the territories, except for Prince Edward Island, the Northwest Territories and Nunavut were interviewed. Interviews were from 45 to 90 minutes in length. The word count of the transcripts ranged from 1687 to 14, 688 words, with an average of 7, 277 words. The total word count was 291, 093 words. Participants lived in diverse geographic population centres ranging from large to small urban and rural centres. All children had at least one NDD with a diverse range of diagnoses represented. Fifty five percent of the children did not have a previous diagnosis of an emotional, psychological or mental health condition. Table 1 describes participants’ characteristics

**Table 1.** Demographics of Participants

Characteristics	Number of Participants	Percentage
**Language of Interview**		
English	34	85.0%
French	6	15.0%
**Gender Identity of Participant**		
Male	2	5.0%
Female	38	95.0%
**Gender Identity of Youth with NDD**		
Male	28	70.0%
Female	12	30.0%
**Age of Participant**		
18–29 years	1	2.5%
30–39 years	9	22.5%
40–49 years	16	40.0%
50–59 years	11	27.5%
60–69 years	2	5.0%
**Age of Youth with NDD**		
0–5 years	3	7.5%
6–10 years	12	30.0%
11–15 years	9	22.5%
16–20 years	9	22.5%
21–25 years	3	7.5%
26–30 years	2	5.0%
**Relationship of Participant to Youth with NDD**		
Biological Parent	29	72.5%
Adoptive Parent	9	22.5%
Other Relation	2	5.0%
**Indigenous Self-Identification**		
First Nations Status	1	2.5%
Métis	4	10.0%
Non-Indigenous	33	82.5%
**Educational Attainment of Participant**		
High School Graduation	1	2.5%
Some College or Technical Training	5	12.5%
College or Technical Training Graduation	13	32.5%
Some University	4	10.0%
University Graduation	16	40.0%
**Gross Household Income in 2018**		
<$40,000	4	10.0%
$40,000-$79,999	15	37.5%
≥$80,000	17	42.5%
**Community Type**		
Large Urban Population Centre (100,000 or more people)	18	45.0%
Medium Population Centre (30,000 and 99,999 people)	9	22.5%
Small Population Centre (1,000–29,999 people)	11	27.5%
Rural Area (<1,000 people)	2	5.0%
**Province/Territory of Residence**		
Alberta	5	12.5%
British Columbia	4	10.0%
Manitoba	7	17.5%
New Brunswick	2	5.0%
Newfoundland and Labrador	1	2.5%
Nova Scotia	2	5.0%
Ontario	4	10.0%
Québec	7	17.5%
Saskatchewan	7	17.5%
Yukon	1	2.5%

Below is a description of the main themes and implications for practice that were identified by participants.

### Implications of denials, delays, and disruption from COVID-19 measures

**Figure 1.**
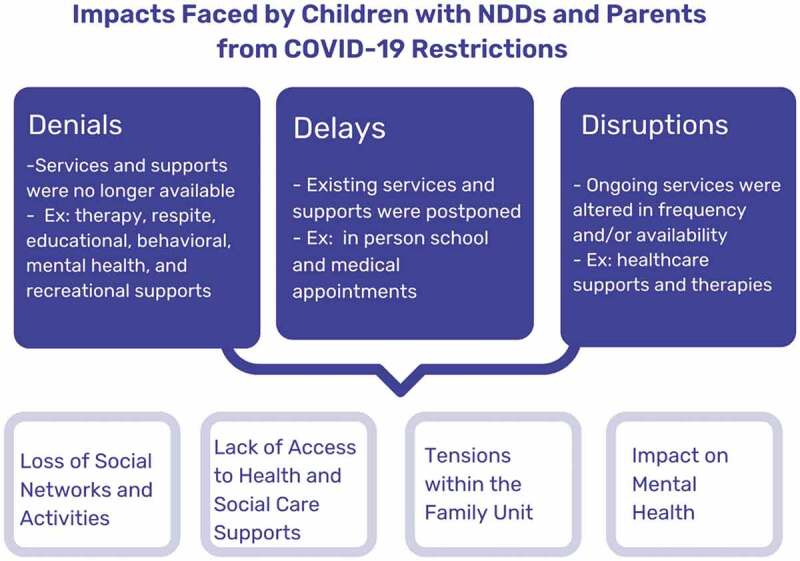
Impacts Faced by Children with NDDs and Parents from Covid-19 Restrictions.

The participant descriptions of their experience of parenting and caring for their child during the pandemic were organized into three overarching themes (Figure 1). These three themes (denials, delays and disruptions) became a way to classify the type of restriction for parents and children. Four sub-themes were identified within these three overall processes. The first two sub-themes focus on changing conditions brought about by restrictions and the last two discuss the impacts of these conditions on mental health and well being:1) loss of social support and activities; 2) strain in access to health, education, and social care support; 3) tensions within the family unit; and 4) impact on mental health.

### Loss of social networks and activities

Parents described having to manage their children’s care in the home with few supports from others. Participants lost established social networks and support systems during the pandemic with restrictions on social gatherings. Parents of children with NDD could not rely on others to assist with managing care needs and associated behavioural challenges that may have been exacerbated during the pandemic due to restrictions on social contact. One parent commented on the physical work, *“my son is 24/7 care and all of the extra work that goes into that”* and emotional effects from losing social care support within the home, *“ … you don’t get a break.”* (Parent #19). In-home support from extended family members was also lost because of pandemic restrictions. This is described by a participant:

*“ … having a child with a disability, um, we require support from family to help, to help care for [child’s name], …… . We’re unable to do that due to lockdowns and restrictions, so the isolation through the pandemic and lack of family support made things very difficult.”* (Parent #38)

Families also noted experiencing isolation and loss of social connections as extended family members and friends could no longer visit in the home. This lack of support was common among participants. One parent stated,

*“Our social life fell off to nothing, family support fell off to nothing. … what is difficult in our family is the impression of repeating the same thing every day, day after day, with no real objective. We have the impression that it will be like this until the end of time, and that is hard. It is heavy to carry.”* (Parent #20)

Participants often noted that their children could not understand why they could not see the people they cared about because of COVID-19 policy measures. As one parent said, *“He doesn’t get why, his favorite auntie who lives just across the street can’t come over visit,” (Parent #19)* and another parent said, *“He thinks I’m just being mean and not letting them to go to grandpa’s house”* (Parent #22).

Parents also expressed that social isolation and lack of support contributed significantly to mental health challenges. A parent shared,

*“ … feeling isolated for me-I was so lonely. Like, I’m a single mom-with two special needs kids-and, it was so hard. My mental health crashed and burned. I couldn’t find the motivation to do what I needed to do. I couldn’t join the Zoom classes.”* (Parent # 34)

Parents also highlighted the loss of social networks and social skills for their children with disruption and denied access to community activities and attending in-person school.

*“ … [T]hat’s probably the … biggest area in our lives that has been … impacted … . [Child’s name] was … really impacted with … losing his friends. So because it was like a loss to him, it was … like not seeing his teachers anymore. ‘Cause he just loved his school. They were so supportive of him and his Tourette’s and our family.”* (Parent #22)

Several parents commented on restrictions when visiting their children in supportive living arrangements and the effect this had on their children’s mental health and behaviours. As one parent shared,

*“My 15-year-old lives in a support home because he can’t live at home anymore. And I know it’s been particularly hard on him. Because we’ve had less visits because it’s just not safe for visits. So, his mental health has definitely taken a hit. And he has cut and been suicidal during this time. And required additional support from his support people.”* (Parent #23)

And another parent shared the inability to visit her daughter in a group home. *“She does not understand why mummy and daddy are no longer going to see her”* (Parent #10).

Participants also noted major denials in access to community facilities and activities that had provided supports to the family prior to the pandemic such as after school and recreational programmes, libraries, museums, and community pools. Families had relied on these supports for structured activities for their children with NDD as other types of recreation were not available to their child with their physical or emotional limitations. There were generally no replacements for these community activities for children. One participant commented on the loss of overall community services, *“there was a total lack of services at first- … uh, that was very, very frustrating. That was very, very stressful … for us … as a family.”* (Parent #33). Support was hard to find pre pandemic and even harder for the family to access during the pandemic. *“We have always had trouble accessing services for him and COVID-19 just made it a thousand times worse.”* (Parent #3).

Physical activities had previously helped children release and manage the anxiety correlated with their NDD diagnosis. Parents struggled to find activities their children could participate in. One parent commented on the limited activities available,

*“I mean, if we’d been able to have something that we would have been comfortable with our son doing that might’ve been able to help with his anxiety issues and then in turn help with his actual physical health issues, that would have been good. But I don’t think that there actually was, or even is anything that would have existed.”* (Parent #37)

Another parent commented on the loss of recreational programmes,

*He has no coordination … he can’t, um, he can’t skate, he can’t play hockey, he can’t, you know, do other things that other, like, kids could do out, go outside on the street … he can’t do … . so there’s nothing for that child … where do you let him go … gyms are closed … . so he sits because that’s all he knows how to do, right? … he sleeps … a lot. And so then, yeah, that’s where the depression comes in and the anxiety of, like, what’s tomorrow gonna bring?* (Parent # 36)

These restriction in recreational opportunities contributed to families being confined to their homes, particularly in the winter months. One parent discussed an increase in challenging behaviours from her child from these confinements, “*So, he was very stressed. Um, and anxious and angry, and frustrated, and- … not sleeping, ‘cause, you know, like, we were almost, like, trapped in the house. It was- it was ugly there, for a while.”* (Parent #12)

Parents expressed how the loss of activities, reduction in family and friend support, feeling trapped at home, and the endless disruption of routine led to behavioural manifestations. Children experienced increased depression, sadness, self harming behaviours, and anxiety. Two parents shared the impact of the loss of these activities on their children’s mental health. One parent said, *“So when the pandemic started last year … [child’s name]’s anxiety skyrocketed*.” (Parent #22) Another parent said,

*“ … last summer, um, was very, very harsh for her because like I said, everything was changed and, you know, it was very different. And so she had a lot of, you know, hitting herself- … and, just a lot of crying, upset all the time, very emotional- um, you know, confusing for her. So it was very, very challenging for, for us to try to figure out what’s wrong.”* (Parent #18)

### Strains in access to health, education, and care supports

Families also experienced strains in accessing medical specialists, therapists, respite workers, childcare, school aides, teachers, and mental health supports. Participants expressed frustration and worry that their children could not access the services they needed, and the onus was on parents to find services when they are already managing caregiving, work, school, and other obligations. Several parents discussed the lack of access to medical providers during the pandemic to support parents with managing pre-existing mental health challenges:

*“Super frustrating. You know, like when you are seeking support for something that, you know, that can be resolved by, you know, seeing a psychologist or a specialized doctor, and you can’t get access to that, it’s extremely frustrating. And not to mention, it’s just, it’s just not right. You know like you’re, you’re, you’re making a child suffer for uh, you know, a reason that’s beyond out of your control, like you just can’t get help. Like everywhere you turn, there’s nowhere to go, you know, you just can’t get any help.”* (Parent #5)

Another participant provided an example of the loss of the nurse practitioner with no virtual support in the initial closure of community medical facilities:

*“Even though their symptoms or their depression or their anxiety has increased the need for medication, it [medication] wasn’t able to be increased because we didn’t have, we didn’t have that contact with our nurse practitioner. Um, and then when we weren’t allowed in the hospitals, you know.”* (Parent #36)

Parents also discussed their need for community respite in the home and community settings before the pandemic to replenish their capacity to care for their children. Respite and in-home health care support was not available for months to a year for some families. One parent expressed her frustration with lack of respite supports,

*“We have nothing. Nothing. Because everything is closed … So no, the proposals made were really not suited to our needs. … What was really hard for us was not having any respite and not having a life raft we could throw ourselves on when we were at the end of our rope. So yes, it had an impact. COVID-19 had an incredible impact on our capacity to withstand, on our mental health.”* (Parent #29)

Parents were also responsible for school supports when school programmes and activities transitioned to distance learning as their children usually required assistance from an aide or other supports in the classroom. Several parents commented on the additional responsibility to look after their child’s educational needs at home in combination with the physical and emotional needs, and its effect on the parents’ mental health in addition to the child’s: *“The assumption during COVID-19 was that parents would be at home providing the physical and emotional and educational support at home* … ”(Parent #38) and another parent said, *“I’m supposed to work full time, homeschool a child with significant learning challenges who can’t learn on their own. Maintain employment, maintain sanity”* (Parent #39).

Also returning to in-person school after pandemic closures and lockdowns, with online learning or no supplementary learning, was a source of anxiety for many children with NDDs. In some cases, COVID-19 restrictions in schools did not consider the needs of children with NDD. One parent discussed that the restrictions did not allow her to accompany her child into the classroom:

*“ … now that he’s back at school, we have a little bit of a separation anxiety issue, I think. … ‘cause last year [prior to the pandemic] I could actually walk him into school and walk him into his classroom and get him settled and he’d know that everything was okay, but now they’re … everybody’s got a lineup outside of the school and the teacher just brings the whole class in at once. And he gets upset pretty much every morning.”* (Parent #37)

Another parent commented on changes that were made in the classroom cohorts with little consideration for the needs of children with NDDs and the mental health implications for her daughter:

*“The kids can only stay with certain kids. It’s like, they’re only with their grades. Well, when I have a kid that’s developmentally younger than her peers, school has been so hard this year because she’s not developmentally where her peers are at. So, her peers, like they’re asking her at school, if she’s retarded you know. And so then compiled with the mental health piece, and the suicidal thoughts … so it’s, it’s hard and pre COVID, at least she could socialize with the younger kids and that seems to help a bit. And so now that’s, I would say another piece of the COVID piece.” (*Parent #39)

Parents also expressed the lack of mental health support for families and children during the pandemic when they lost established routines, schedules and supports. A parent shared it was a crucial time for mental health supports that were no longer available,*“In a time when mental health supports were completely yanked out from under us, we all needed more.”* (Parent #34). Parents also relayed that mental health supports that were available had long wait lists. There were few mental health supports to address urgent concerns for children with NDDs and their family members during the COVID-19 pandemic. As one caregiver said,

*“there’s all types of programs, but it’s not that easy to get into those programs. It’s not something where you could go up and say like, I need help and you’d be taken care of right away.”(Parent #7)* And another parent said, *“The wait-time is six months or a year, but by that time it is no longer relevant. The need is immediate, when you want to call a psychologist”* (Parent #20).

Many parents shared that if they needed mental health support for their child, they would not know where to find these supports. Others commented that with their child’s unique challenges from their NDD, and lack of available mental health supports, there was no one to address their child’s needs. Parents questioned if accessible and inclusive mental health support for their children with NDDs (for example, children with low communication abilities) even existed:

*“But how do you deal with anxiety in a child and children who are prone to anxiety? Like, how do you, you know, how do you find somebody who’s got that specialized, you know, and not just anxiety, but like … how do you help a child with anxiety who also has communication challenges?”* (Parent #26).

Some parents could also not find mental health supports for themselves during the lockdowns. A parent described her inability to access mental health support and how this diminished her capacity as a caregiver: “*I’m a caregiver. I look after everybody else but myself, you know? So, I’ve lost those support services [referring to psychiatric services] through this as well.”* (Parent #31).

### Tensions within the family unit

Parents also described how restrictions and lack of services and supports led to tensions within the family unit. Children with NDD and their families experienced a significant loss of stability, certainty, and predictability, which impacted the functioning of the family unit. As a parent stated: *“ … so it is very different. Especially for a child with autism that- … you know, thrives with, um, you know, sameness … they want routine and you can’t provide that for them.”* (Parent #18). These essential structures and supports had been established and developed by the family over the course of the child’s life. Another parent concurred and said:

*“Routine is very important, and her need to be able to go to school or … and be able to do physical things, like get out of the home- … . go to daycare and be able to exert physical energy through play and doing things … without her able to do that, her disability … she had increased frustration. Uh, she lashed out more against her brother.”* (Parent #38)

Parents had the responsibility to try and manage the change in routines and supports. One parent caring for a child with autism discussed the impact on the child and family,

*“I mean, we have so many things to manage – the routine. Yes, we had to adapt, we had to find tools to reduce the stress, reduce the autistic crises, reduce a bunch of parameters that we did not have to manage before because we had a routine that was already set up. So it had a very, very big impact on us. And, um, and the rest of us were impacted by that mentally very much, yes.” (*Parent #29)

Parents also described a limited capacity to manage everything by themselves when they were already drained before the pandemic from the care needs of their children. A caregiver commented on her inability to handle the daily responsibilities of her son’s care needs:

*“I am gonna say based on my son’s needs, we operate on a pretty constant state of exhaustion anyways. Like he requires constant supervision. So when you’re kind of operating out of that baseline, it doesn’t take much for things to change or fall apart kind of quickly.” (*Parent #31)

Another parent expressed her frustration at the lack of support to navigate all the changes and its impact on parental capacity.

*“Um, but I think it’s more frustration because it’s, like, where- where do you go from here? Like, how do you navigate this? And, uh, and there’s no manual. There’s no manual that tells you, “Oh, this is how you should do this during a pandemic.”* (Parent #25)

### Impacts on mental health

The continual loss of routines and structure, regular activities, and limited supports and services had debilitating mental health impacts on the individual with NDD and their families. With continual denials and disruptions to essential family supports, children experienced mental health challenges such as anxiety, depression, sadness, stress, frustration, loneliness, and sadness. The drastic changes of having support before the pandemic to not having any support also impacted children. This parent expressed the mental health difficulties for her child,

*“Um, so I think it was a combination of like, for a kiddo that’s like fully supported, doing okay. And then we go back to supports the whole world changed. Right? And it just kinda escalated the, the mental health piece for her. Like to the point where she was, she couldn’t be left alone. Like there was, there was, yeah there still is worries of self harm and like hallucinations, et cetera. Like they weren’t just minimal, they were pretty extreme.”* (Parent #39)

Some children experienced a worsening of challenging behaviours associated with their NDD from social isolation and no access to other support. Challenging behaviours included anger, frustration, aggression towards others, aggression towards self, refusal to do activities, and rigidity in how activities were done. One parent expressed an increase in challenging behaviours due to social isolation:

*“I mean, starting with [child’s name], already having mental health struggles, um, behavioral struggles that add to the mental health and then just being shut down completely from everything. And even for myself being so sequestered, just him and me … ‘cause that’s FASD and ADHD cause him to be quite aggressive and physical with me quite often so I had that whole thing as well as dealing with like, I was literally living in a domestic violence situation with a child- … and I couldn’t do anything about it.”* (Parent #3)

Some children also experienced an increase in behavioural issues from COVID-19 from mask wearing and social distancing. Parents commented that their children with NDD had difficulty complying with certain public health measures: “*He doesn’t like things touching him”; “Putting a mask on their face, that is hell. Pure hell. [Child’s name] does not go out much because of that”* (Parent #9). Some children were not able to continue with regular activities because of these sensory issues and became more isolated. Other children refused to leave the house and take part in outdoor activities because of discomfort with physical distancing restrictions. As one parent relayed: *“And a lot of times my son was having a harder time just being flexible during the pandemic. It was, for him very difficult. So, a lot of times he didn’t want to go out and do things.”* (Parent #28)

Over different phases of the pandemic, community, medical, and school support and services were closed or reopened for short periods of time to then abruptly closed again. One caregiver expressed the difficulty for children with NDD: *“It was especially hard for, [child’s name] just ‘cause it’s all like … it’s just constantly changing. Um, so it’s kind of hard for him to keep track of what he can and can’t do and where- where we can and can’t go”* (Parent # 37). Families did not know if their children were going back to school and how long they would be in school once it reopened. Children frequently went from face-to-face instruction at school to remote learning instruction and back again to in-person school. One parent described the anxiety for her child with uncertainty of school closures,

*“ … she’s wanting to go back to school. And now you’ve got to explain to her, well, you might not be going back to school. It was just so up and down and just- … so it is very different. Especially for a child with autism that- … you know, thrives with, sameness and, you know, they want routine and you can’t provide that for them.”* (Parent #18)

As well, the transition of going back to school after being away from school, increased anxiety, and physical symptoms of the child with NDD: *“You have to repeat the transitions and repeat the social scenarios … you have to repeat everything. Every time they go back, after a long period at home, the child is disturbed. So, you have to start all over”* (Parent #29).

Parents also expressed a regression of their child’s social skills and increase in social anxiety from limited social connections with peers and adult with school closures during different phases of the pandemic.

*“You have to learn it all over again. You have to start all social scenarios at zero. There is a mechanism that you have to launch and it is exhausting because when your child has a crisis, there is often, as you know, a complete regression. So everything you worked on for years, pffft, it’s as if it never happened*. (Parent #29)

Conversely, two caregivers commented on positive impacts to their children’s mental health because of restrictions brought in at different times with the COVID-19 pandemic. One parent expressed there was less bullying with her child as she was not in school: “*Um, we actually had a positive experience because when she was in school, she was bullied*” (Parent #14). Another parent discussed his child’s reduction in social anxiety from doing schoolwork at home and not in the school environment: *“He loved the online learning for the past two weeks because he was able to be at home and didn’t have to be in school, you know, worrying about and having anxiety”* (Parent #22).

Parents also experienced anxiety, stress, feeling overwhelmed with few supports, and overall worry about each member of the family, in managing their child’s care during the pandemic. One parent discussed the impact of these denials and disruptions on their family: *“I’m gonna say definitely an increase in stress- … for, you know, everyone involved. Um, having a child who’s not doing well … because his supports are pulled away … definitely was a major cause of stress for both me and my husband”* (Parent # 11). Another parent commented on this experience of worry and frustration and impact on the overall functioning of the family with so many changes and transitions to navigate:

*It’s sadness and not knowing, and I think I worried about [child’s name]’s health, worried about my husband’s health. And I started to feel like, a couple weeks ago, was just that I was just this bad mom who couldn’t … get through this. And I, and I, and I understand that other parents are probably going through this too, and they’re, they’re feeling all helpless* (Parent # 40).

The lack of medical and social care supports for children with NDD compromised parental mental health and led to increased stress, anxiety, loneliness, depression, and exhaustion for parents. As one parent said, *“I can’t do this anymore. Like, I, I’m falling apart”(Parent #* 23). And another parent talked about the impact on mental health for herself because of reduced capacity,

*I think that people’s mental health is getting to a point where we’re, we’re heading into doomsday. How many people are gonna commit suicide over COVID? Like, how many people are gonna need to see psychiatrists over this and to get through life? And I think I am one of them […] ‘I’ve gotten more anxiety having to stay in the house* (Parent #40)

Some parents experienced worsening mental health issues with the pandemic because of the strain and limited capacity to handle everything on their own.

*“I’ve had anxiety issues for a while, but they’ve definitely spiraled a little bit, I guess you could say. I mean, going to the grocery store … is an ordeal. My husband as well, like he’s had mental health issues. I mean he did before, but it’s definitely gotten a lot worse* (Parent #37).

## Discussion

The findings from this study provide important insights into the struggles faced by families during the pandemic. Mental health challenges were experienced by parents and children with NDDs with extensive COVID-19 restrictions implemented across Canada and the absence of alternative measures to support families and children in response to these restrictions. These findings are in alignment with studies which have described an increase in mental health challenges experienced by families of children with NDDs (Bentenuto et al., 2021; Breaux et al., 2021; Cluver et al., 2020; Gadermann et al., 2021; Lee et al., 2021; Patrick et al., 2020; Shorey et al., 2021; Yusuf et al., 2022). Our study further explored caregivers’ perspectives on the impact of COVID-19 policies and restrictions on their child and family experiences during the pandemic.

### Drawing lessons for future emergency preparedness

The participants in our study experienced significant denials, delays, and disruptions in service and support provisions that resulted in the perception of mental health challenges. The absence of population specific measures to ensure that these disruptions did not have differential impact on vulnerable groups was also impactful. This study demonstrated the need for population-specific measures for vulnerable groups to ensure that necessary public health measures do not cause unintended harms.

International agencies such as the WHO and UNICEF have developed specific guidelines and recommendations for considering the specific needs of persons with disabilities during public health preparedness and responses (UNICEF, 2021; Thompson Burdine et al., 2020). There is an opportunity for thoughtful and meaningful disability inclusive support and services for families with diverse needs for future emergency preparedness. Many of the systems and supports families of children with NDD depend on, did not have emergency preparedness strategies, resulting in denials, delays, and disruptions in supports and services. The loss of social, educational, and therapeutic supports for children with NDDs was detrimental and led to mental health impacts. Future emergency preparedness planning in public health or any emergency, needs to consider a disability inclusive approach allocating appropriate resources for family supports in the home and community to meet the needs of children with NDDs and their families (Hoover et al., 2022). Our findings demonstrated a need for consideration of this planning in several different sectors including respite care, recreational services, home care, community health, school, and mental health services.

Many parents shared that one of their biggest unmet needs during the COVID-19 pandemic was the inability to access respite care. Respite needs to be considered an essential service in providing continuation of care for children with complex health needs at home, as preconized in the UN Convention on the Rights of Persons with Disabilities (United Nations, 2016) As well, respite would be helpful for families with the reintegration and transition of children back into schools and society during different phases of a public health crisis because of dysregulation with disruptions in routines for children (Colizzi et al., 2020; Edelstein et al., 2017; Nonweiler et al., 2020). In addition, families need to be able to visit and support their children if they are in the hospital or care homes and this needs to be considered an essential human right as per United Nations CRPD international commitments (United Nations, 2016).

Families communicated a loss of connection and isolation from social and medical care supports. Parents would have benefited from behavioural support for their children by telephone or with virtual meetings for the management of heightened anxiety and dysregulation that comes with a public health crisis (Summers et al., 2021). Parents would have also benefited from regular places of connection and check in points from school therapists, teachers, family physicians, psychiatrists, psychologists, and community support workers (Cortese et al., 2020; Fazzi & Galli, 2020; Narzisi, 2020). These check in points could include accessible support through electronic communication, phone calls, and virtual meetings (Fazzi & Galli, 2020). As well, telehealth and telerehabilitation with medical specialists and other therapists could be used to increase therapeutic and medical support for families (Camden & Silva, 2021; Cortese et al., 2020; Trabacca & Russo, 2020; White et al., 2021). One example of this occurred at the Hospital for Sick Children (Sick Kids) in Toronto, Ontario, Canada, where a multidisciplinary clinic was offered for families caring for children with NDDs during the pandemic. This clinic provided family centred support and solution focused care through virtual consultation (Summers et al., 2021).

Schools and the education sector also lacked disability inclusive emergency preparedness planning. The loss of structure and routine of in-person learning increased mental health effects in children with NDDs and added stress and tensions to families as described in other global research studies (Asbury et al., 2021; Lee et al., 2021; Viner et al., 2021). Online instruction must be adapted for children with NDDs. Distance learning requires the use of diverse learning modalities and pedagogies for children who learn and communicate in different ways. There needs to be recognition that some children and youth with NDDs cannot manage the technology in remote learning and may disengage with learning and communication challenges (Cortese et al., 2020; White et al., 2021). In addition, parents also described experiencing mental health challenges from coordinating care for their children, along with working from home, and offering support for remote learning (Daulay, 2021). Individual educational supports must be offered to families in the home or with remotely scheduled touch points for short periods each day for routine and predictability, as well as relieving parents of the responsibility of providing curriculum instruction (Kang et al., 2020; Toseeb & Asbury, 2021). These touch points could also provide much needed social connection and social support for children and families (Guller et al., 2021; Toseeb & Asbury, 2021).

The increase in mental health concerns during the pandemic for both children and parents contrasted with the limited access to mental health supports and programmes for daily living such as recreational and community programmes. The need for these supports became evident and critical during the pandemic. This study highlighted the need to develop and share information about these programmes for families from government sources or existing community health centres so that families can easily access them in time of need (Degli Espinosa et al., 2020; Esentürk, 2020; Narzisi, 2020; Strudwick et al., 2021).

Mental health supports should also be part of the basic infrastructure offered for families and youth with NDDs. Anticipating that children with NDDs and their families may be experiencing new of worsening mental health symptoms, maximum amounts of support in times of uncertainty with a public health crisis should be available and accessible. Services could include collaborative networks of experts such as counsellors, psychologists, psychiatrists, researchers, and community mental health support workers with training in working with youth with NDDs and their families (Edelstein et al., 2017; Liu et al., 2020). Access points within collaborative networks for families could offer 24-hour hotlines, manned nationally and provincially, as well as call centres, and community mental health clinics with virtual and in person appointments (Fazzi & Galli, 2020; Liu et al., 2020). Access to information about the public health crisis and precautionary measures must be tailored for children with diverse needs and provided for families over the internet, text, and call centres, with the use of visuals and simple messages that can be understood and followed by all.

Adaptations to mental health supports was seen as a viable alternative for parents, in the absence of formal, structured supports. Informal supports such as social connections and social networks is one alternative that can be promoted to reduce mental health burden. This may include using social media platforms (and subsidizing access to platforms) with extended family members such as grandparents and close friends to engage in video calls. Children could connect with close friends on play dates over a social media platform with a virtual call (Kang et al., 2020), or engage in online leisure activities such as sports, arts, or story time (Yoo et al., 2022). Parents could also benefit from online social and educational support from other parents with NDDs who share similar experiences (DeHoff et al., 2016). Costs for technology need to be supported for families in financial need to reduce the impacts of social isolation and challenges with internet connectivity in rural settings (Shorey et al., 2021).

Parents also expressed that the lack of schedules and routines was detrimental for their children and family life. Supportive coaching with parents to develop new structured daily schedules for children can be developed. Examples of such initiatives were reported in Italy, USA, and China (Cortese et al., 2020; Degli Espinosa et al., 2020; Kang, et al., 2020; Liu et al., 2020). Coaching can also include apps and web-based resources for ease of access for families (Moreno et al., 2020; Strudwick et al., 2021).

### Moving forward with a COVID-19 recovery plan

This research provides important context to inform a focus on a recovery plan to reduce further mental health challenges as the world emerges from the pandemic. The Federal and

Provincial/Territorial Governments of Canada have recognized the need for an inclusive COVID-19 recovery plan, a strengthened healthcare system and public health supports “for all Canadians, especially seniors, veterans, persons with disabilities, vulnerable members of our communities, and those who have faced discrimination by the very system that is meant to heal.” (Speech from the Throne, 2021, page 10). As a signatory to the UN Convention on the Rights of Persons with disabilities (United Nations, 2016), Canada has committed to policies and programmes to reduce barriers to participation in society. Post pandemic recovery strategies need to be guided by this rights-based framework and the UN Research Roadmap to Post pandemic recovery (UUnited Nations, 2022). Key relevant pillars in this strategy include social protection and basic services, health systems and services, social cohesion, and community resilience. Nation wide supports that are aligned with these pillars will help to mitigate the denials, delays and disruptions from pandemic emergency responses and associated mental health challenges

With a federal and global emphasis on public health support for persons with disabilities, efforts must also be made within provincial, territorial and local governments towards a disability-inclusive COVID-19 recovery plan, with mental health support. Knowledge from our study can inform mental health recovery strategies and family centred interventions to minimize further pandemic disruptions to children and caregivers’ mental health. Alignment with the priorities outlined in these rights frameworks and strategies, requires the voices of families and youth with NDD in the identification of gaps and solutions in COVID-19 recovery policies while enhancing mental health supports and services in communities and institutional care. These supports must also consider the voices of parents who experienced substantial mental health challenges from the increased responsibilities during the pandemic (Gadermann et al., 2021; Shorey et al., 2021).

Establishing sustainable approaches for rapid consultation is a critical component of developing disability inclusive supports. There was concern from parents in our study that public health policies and government decisions did not consider the diverse needs of persons with NDDs by utilizing a disability inclusive perspective when mandating school, health and community agency closures and lock down measures. System, services and emergency response strategies must be co-developed with stakeholders including families of children with disabilities and youth with disabilities themselves. Parents spoke of wanting to have a voice in decision making and a contribution in the determination of policy restrictions that impacted their children in the community. Disability advisory groups could be established across Canada and include participation from families in both rapid consultation and the design and recommendations of an integrated mental health system (Krahn, 2021; Subramaniam & Villeneuve, 2019). This integrated system must be readily available, flexible, and adaptive in the provision of mental health support for children and parents in communities, to manage new or worsening mental health challenges from COVID-19 in Canada (Carr, 2014; Moreno et al., 2020).

## Strengths and limitations

The strengths of the study include an increased understanding of the impact of COVID-19 on parents and children with diverse NDDs across Canada from COVID-19. As well, forty parents gave rich descriptions of the impact of policy restrictions on their everyday lives which ultimately affected their mental health. There are three limitations in this study that could be addressed in future research. First, the cross-sectional design of this study meant that we captured participant perspectives at one point in time, specifically during the third wave of the COVID-19 pandemic. As such, we may have missed perspectives relating to other phases of the pandemic. Secondly, in light of significant mental health challenges for children with NDDs and their parents, longitudinal studies exploring the long-term mental health effects requires further research. Thirdly, although maximum variation sampling was used and recruitment support was provided for underrepresented areas, more representation would have added to the diversity of the sample from isolated and remote communities such as the Northwest Territories and rural areas, Francophone speaking parents, and male caregivers. Notwithstanding limitations in some areas of data representation, results from participants across interviews were consistent and alternative responses were included in the data.

## Conclusion

Parents and children with NDDs experienced mental health challenges because of denials, delays, and disruption of support and services during the COVID-19 pandemic. Many of the challenges resulted from necessary public health measures with unintended harms for sub-populations like families with children with NDDs. This study can inform a disability inclusive COVID-19 pandemic recovery plan and future emergency preparedness planning to mitigate adverse mental health impacts. Learnings from parents caring for children with NDDs can also be considered by local and national stakeholders to improve access to support and services for families moving forward.

## APPENDIX 1: COVID-19 INTERVIEW QUESTIONS

Part 1: Access to Services During the COVID-19 Pandemic

For the first part of the interview, I will be asking you questions regarding your access to services and supports during the COVID-19 pandemic.

1. Has your access to the services/supports for your child with a disability changed since the start of COVID-19 pandemic? If yes, how has access to services/support changed? Can you reflect on how your access to services and supports has changed as the pandemic has progressed (from the initial lockdown phase, to the re- opening phase, and to the current second wave phase)? Explain.
  - Additional Probes:
    - What services/supports do you use, and how have these changed?
    - How has the delivery of the services changed during the pandemic? For example, have you accessed any services or supports virtually, over the phone, or using apps?
    - What services/supports were easier/harder to access during the pandemic?
    - Did anyone help you access services/supports? If yes, how did they help you?
    - What did you need the most/least from service providers, educators, and/or physicians?
    - Were there services/supports that were cancelled due to the COVID-19 pandemic? Were there services that you were accessing before the pandemic that you are no longer accessing? How has this impacted you?
    - For parent/caregivers of children in school and youth in school/university: How have school closures during the COVID-19 pandemic impacted you/your child? Have school closures impacted your access to services? Have schools adequately supported your/your child’s learning during the COVID-19 pandemic? How have/has you/your child responded to online schooling?
2. Have you been experiencing any challenging behaviours or behaviour changes with your children during the pandemic? If yes, how have you received support for these behaviours? Explain.
3. During the COVID-19 pandemic, did you apply for any new services/supports that you were not receiving previously? If yes, what has your experience been applying for these services/supports? Can you reflect on processes that worked well or that did not work well? What made it easier/harder to apply for services/supports?
  - Were there services that you thought about applying for but that you did not end up applying for because you thought COVID-19 might be a barrier to receiving? Explain.
4. Reflecting on the needs of you and your family during the COVID-19 pandemic, to what extent do the services/supports you currently have access to fulfill these needs? Are there other services/support that would be helpful for you and your family? Explain.
5. Do you think gender influences the types of services/supports that are made available for people with disabilities and their families? Do you think service providers perceive people with disabilities differently based on their gender? How have these perceptions about gender and access to services/supports changed or remained the same during the COVID-19 pandemic? Explain
  - Additional Probes:

i. If you identified as (insert other gender options), how do you think this would impact you access to services? What about if your child identified as (insert other gender options)? Explain.

Part 2: Impact of COVID-19 on Family

Next, I am going to be asking you about how the COVID-19 pandemic, and precautionary measures associated with the pandemic, have impacted you and your family.

(6) Has your employment status (or education status), or the employment status (or education status) or your partner/spouse (if applicable) been impacted by the COVID-19 pandemic? Has your employment (or schooling) or the employment (or schooling) of your partner/spouse been impacted by changes to service access during the COVID-19 pandemic? Explain.
  - Has you or your partner/spouse’s employer been supportive during the COVID-19 pandemic? For example, provide flexible working hours to accommodate child care needs, etc.
  - If you or your spouse’s employment (or schooling) was impacted, did you apply for the Canada Emergency Response Benefit (CERB)? If yes, what was your experience applying for and accessing CERB? Did you transition from receiving CERB to receiving EI? What was that process like for you? Explain.
  - Are you or one of your family members a health care or essential worker?
(7) For this question, I would like you to reflect on your health and well-being, and the health and well-being of the members of your immediate family. Have the mental health needs of you or of members of your immediate family changed during the COVID-19 pandemic, or have you or your family members experienced any mental health challenges? If yes, please elaborate.
  - Additional Probes:
    - How do you think the services (or lack of services) you accessed during the COVID-19 pandemic influenced your mental health and overall well- being?
    - Have changes to your mental health impacted your daily routine, or the daily routine of your family? If yes, how so?
    - How has your mental health and the mental health of your immediate family members been impacted by changes to your employment status, or the employment status of your partner/spouse, during the COVID-19 pandemic? (Can use answer to question 5 to probe further)
    - Has worry over finances, food security and/or housing security impacted your mental health and the mental health of your immediate family members? Explain.
    - Have you (or any of your family members) visited or stayed at the hospital during the COVID-19 pandemic? How was that experience for you and your family? Have restrictions in hospitals impacted your mental health, or the mental health of members of your family?
      - If you are comfortable sharing, what was the reason for the hospital visit? Was there an emergency, or were you unable to get in to see your usual health care providers?
    - Have you visited or stayed at a group home or similar care setting during the COVID-19 pandemic? How was that experience for you and your family? Have restrictions in group homes (such as limited visitation) impacted your mental health, or the mental health of members of your family?
    - Are you worried about you or your family members contracting COVID- 19? If yes, how has this worry impacted your mental health and the mental health of your family members?
    - Have you experienced any grief or loss during the pandemic? If yes, how has this impacted your mental health and the mental health of your family members?
    - At what point did you find that things became overwhelming for you? At what point did your mental health needs change significantly?
(8) Have you noticed changes in your mental health, and the mental health of your family members during the different phases of the pandemic (for example, during lockdown periods vs. during re-opening periods)? If yes, how so?
  - Additional Probes:
    - How has gradual return to work and/or school impacted mental health?
    - Has your province/territory entered a second lockdown (or semi- lockdown) phase? If yes, how has the second lockdown period differed from the first with respect to your mental health or the mental health of your family members?
(9) Based on your observation, how have you and your family members (including your child with a disability) responded to the precautionary health measures introduced to curb the spread of COVID-19 (e.g., masks in public spaces, frequent hand washing, physical distancing, quarantining, isolation from friends/family, social cohorts/bubbles, etc.)? How have these measures impacted the mental health of you and of members of your family?
  - Have you had difficulties accessing the supplies you need to follow precautionary measures (ex: PPE, hand sanitizer/soap, disinfectant, wipes, etc.)? How has this impacted you and your family? Explain.

- Additional Probes:
  - Are there any measures in particular that you or your family has struggled with? If yes, how have you managed to address challenges?
  - Rules and precautionary measures have changed continuously throughout the pandemic. How have these changes impacted you and your family?
  - Can you think of any examples of how precautionary health measures have had a positive impact on you and your family? For example: reduced social anxiety, increased family bonding, picking up new hobbies, increased access to professionals due to virtual visits, etc.
  - Have the institutions that introduced these measures been responsive/helpful in helping you with any challenges you have had in adopting the measures? If no, what could institutions do to better help you?

(10) Where do you get information about updates regarding the COVID-19 pandemic and changes to public health measures (ex: news, social media, etc.)? Do you think the level of information you have received has been sufficient (too much, not enough, or the right amount)? Do you think communication about the pandemic and precautionary measures has been appropriate and easy to understand for you and your family? Why or why not?
  (11) What types of activities, if any, have you or your family engaged in during the pandemic to help support your mental health? What types of services and supports would you have liked to have been able to access to support your mental health and the mental health of your family members during this pandemic? Explain.
  (12) Canada has recently approved a COVID-19 vaccine, and vaccine distribution is ongoing. Do you and members of your family plan on getting the COVID-19 vaccine or have you already received the vaccine? Please elaborate.
  a) Do you think governments should prioritize persons with disabilities and their caregivers when distributing the vaccine? Why or why not?
  (13) Is there anything else you would like to share about your experiences during the COVID- 19 pandemic?
